# The surgical technique and initial outcomes of Anatolian neobladder: a novel technique of ileal neobladder after radical cystectomy

**DOI:** 10.1186/s12894-018-0406-8

**Published:** 2018-10-26

**Authors:** Z. Talat, B. Onal, B. Cetinel, C. Demirdag, S. Citgez, C. Dogan

**Affiliations:** Department of Urology, Istanbul University-Cerrahpasa, Cerrahpasa Medical Faculty, Fatih, 34098 Istanbul, Turkey

**Keywords:** Bladder cancer, Orthotopic neobladder, Radical cystectomy, Ileal neobladder

## Abstract

**Background:**

We describe a detailed novel step-by-step approach for creation of an ileal neobladder and compare the outcomes with standart neobladder.

**Methods:**

Between August 2009 and January 2016, 36 consecutive patients with bladder cancer underwent radical cystectomy and orthotopic urinary diversion with an ileal neobladder. A novel technique of ileal neobladder construction, called the Anatolian neobladder, was designed by a single surgeon (ZT). Demographics and clinical data were collected. Perioperative, oncologic, and functional outcomes were reported. Complications were graded as early or late. These outcomes were compared with patients who underwent standard neobladder during this period in our center.

**Results:**

The operation was technically successful in all cases. Early postoperative complications occurred in 33.3% of the patients. Daytime continence was achieved successfully in 83.3% of the patients. No patient had severe metabolic acidosis. Six patients (16.6%) died during follow-up, five due to metastatic bladder cancer and one due to a cardiac problem. There was no any statistically significant difference between novel technique and standard neobladder for oncological and functional outcomes.

**Conclusions:**

The Anatolian ileal neobladder is as feasible and safe as standard neobladder technique for urinary diversion in patients with bladder cancer undergoing radical cystectomy.

## Background

Orthotopic neobladders are constructed for urinary diversion after radical cystectomy. An orthotopic neobladder should achieve several normal bladder characteristics, including a continence mechanism, adequate capacity at a low pressure, and an antireflux mechanism for preventing upper urinary tract dilatation [1].

Camey first described an orthotopic neobladder in which a small tubular bowel segment was used as a bladder substitute by anastomosing it directly to the urethra without detubularization [2]. Since this version was described, many different types of orthotopic neobladders have been described, consisting of different gastrointestinal segments [3–5]. Intestinal detubularization plays an important role in the formation of low pressure and adequate bladder volume. Based on this, various techniques have been acceptable using different types of bowel segments (ileum, ileo-colon, colon, sigmoid) and ureterointestinal anastomosis. Studer et al. reported use of a detubularized ileal pouch as a bladder substitute in patients with an intact urethra after cystectomy [6]. There is no agreement on the significant advantage of a single technique over another, despite the description of each technique by the investigators.

We present our experience with a new surgical technique for ileal neobladder creation using 45 cm of ileum. The neobladder is constructed in a simple triangular configuration without a chimney. A spherical apex segment forms the neourethra at the bottom, which provides a sufficient part for anastomosis. The surgical technique, perioperative complications, and medium-term results, including cancer control and continence, are described. The outcomes of were compared with patients who underwent standard neobladder in our center.

## Methods

### Patients

A total of 52 patients (36 with novel technique, 16 with standard neobladder) with a preoperative diagnosis of invasive bladder cancer who were treated at our center between August 2009 and January 2016 were studied (Table 1). All patients underwent radical cystectomy and urinary diversion with creation of an orthotopic neobladder made of terminal ileum. Their charts were reviewed for preoperative (age, sex, disease status, creatinine level), perioperative (operative time, complications, morbidity), and postoperative (imaging methods, laboratory results, continence, complications, morbidity) data. Patients followed for less than 1 year were not included.

**Table 1:** The preoperative findings

	Novel technique (Anatolian neobladder) (*n* = 36)	Standard technique (Studer neobladder) (*n* = 16)	*p*
Age, years, mean ± SD	54.7 ± 11.6	58.0 ± 4.9	0.16
Sex			> 0.999
-Male	34 (94.4%)	15 (93.7%)	
-Female	2 (5.6%)	1 (6.3%)	
ASA	2 (1–3)	2 (1–3)	0.77
Chemotherapy			
-Neo-adjuvant	9 (25%)	4 (25%)	> 0.99
-Adjuvant	2 (5.5%)	1 (6.2%)	> 0.99

### Measurements

The patients had high-grade recurrent stage T1, T2, or T3 bladder cancer. Preoperative imaging studies, including chest x-ray, and abdominal and pelvic computerized tomography (CT), indicated node-negative disease with no evidence of distant metastases in all patients. We used seftriaxone + metronidazol for antibiotic prophylaxis in patients. Single J stents were removed at postoperative 8th–10th days. Foley catheter was removed at postoperative 21st day after cystogram was performed. Patients were evaluated initially 4 weeks postoperatively and then at three-month intervals. CT and laboratory investigations were performed at each visit. We used routine cystogram postoperative 2. month to see the bladder capacity and check if there is any reflux. Complications were graded as early (grouped based on the modified Clavien system) or late (defined as those occurring more than 1 month postoperatively) [7]. Patients with complications underwent additional imaging studies, including excretory urography, voiding cystourethrography or renal scan, as needed. Continence was evaluated by patient self-report during the follow-up visits and International Consultation on Incontinence Questionnaire Short Form (ICIQ-SF). If the patient uses more than 1 pad per day, he/she is considered to be an incontinent. Urodynamic evaluation was performed in patients with daytime incontinence. The outcomes were compared with standard neobladder [6].

### Statistical analysis

Statistical analysis was done with SPSS for Windows 10.0 (SPSS, Inc., Chicago, IL, USA). Continuous and non-continuous numeric variables were described as median (Range). The Mann-Whitney *U* test was used to compare continuous and non-continuous numeric variables. Kaplan Meier analysis was used to calculate overall survival (OS). *P* < 0.05 was considered statistically significant.

### Surgical technique

**Fig. 1:**
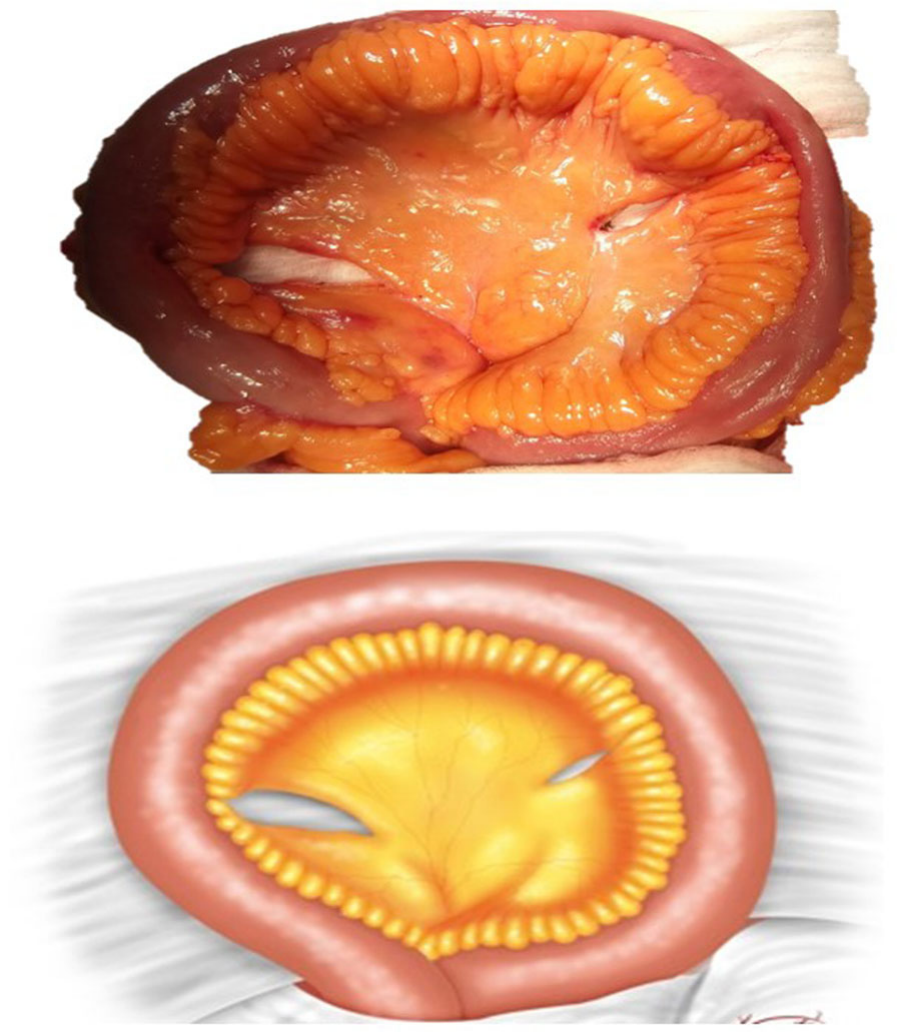
A 45 cm ileal segment was selected, starting at an appropriate point 15 to 20 cm from the ileocecal junction

**Fig. 2:**
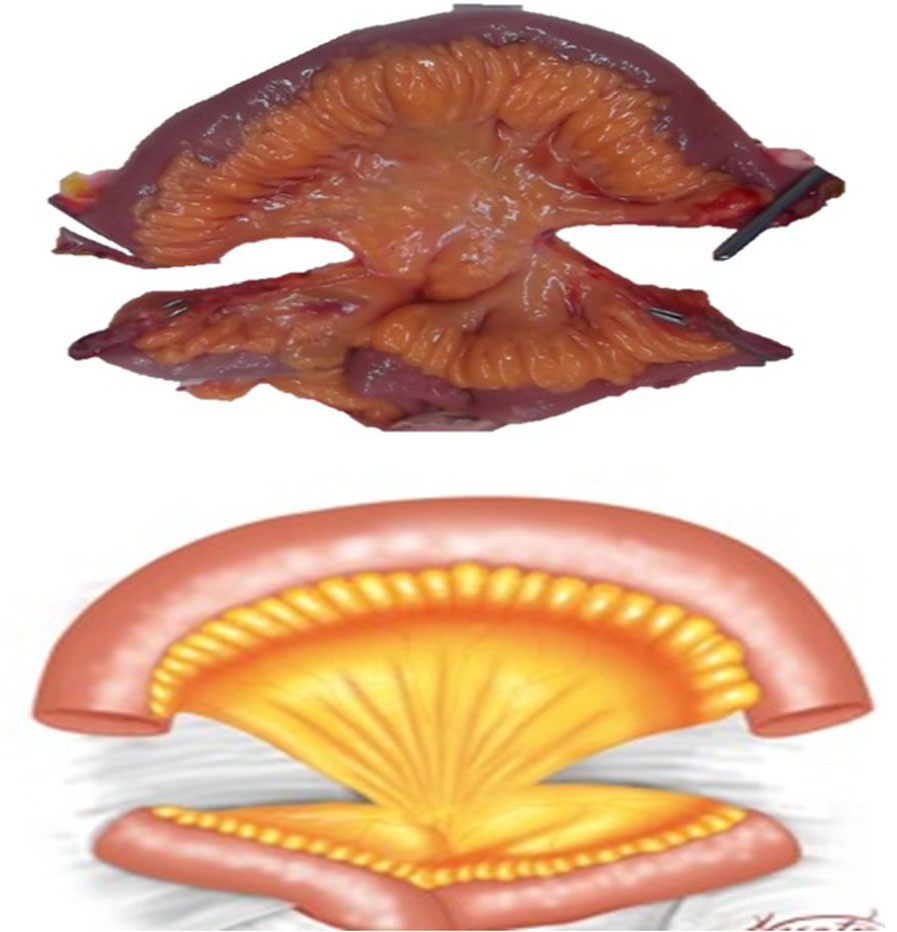
The intestinal continuity is rebuilt by an end-to-end anastomosis with surgical staplers

**Fig. 3:**
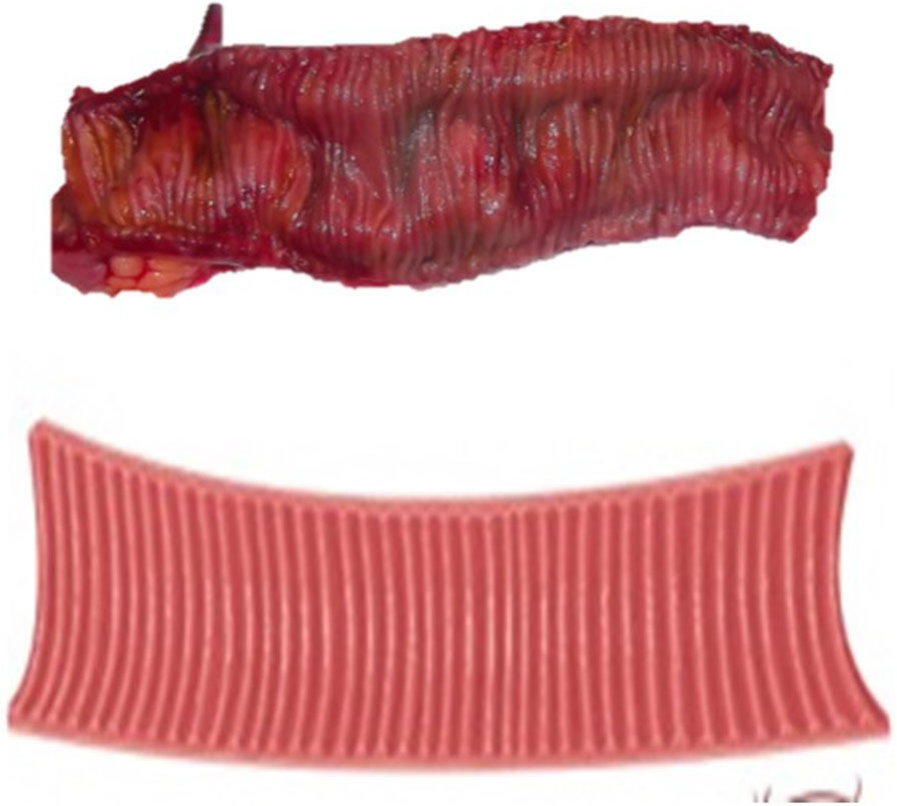
The whole separated ileal segment is cut at the antimesenteric border for detubularization

**Fig. 4:**
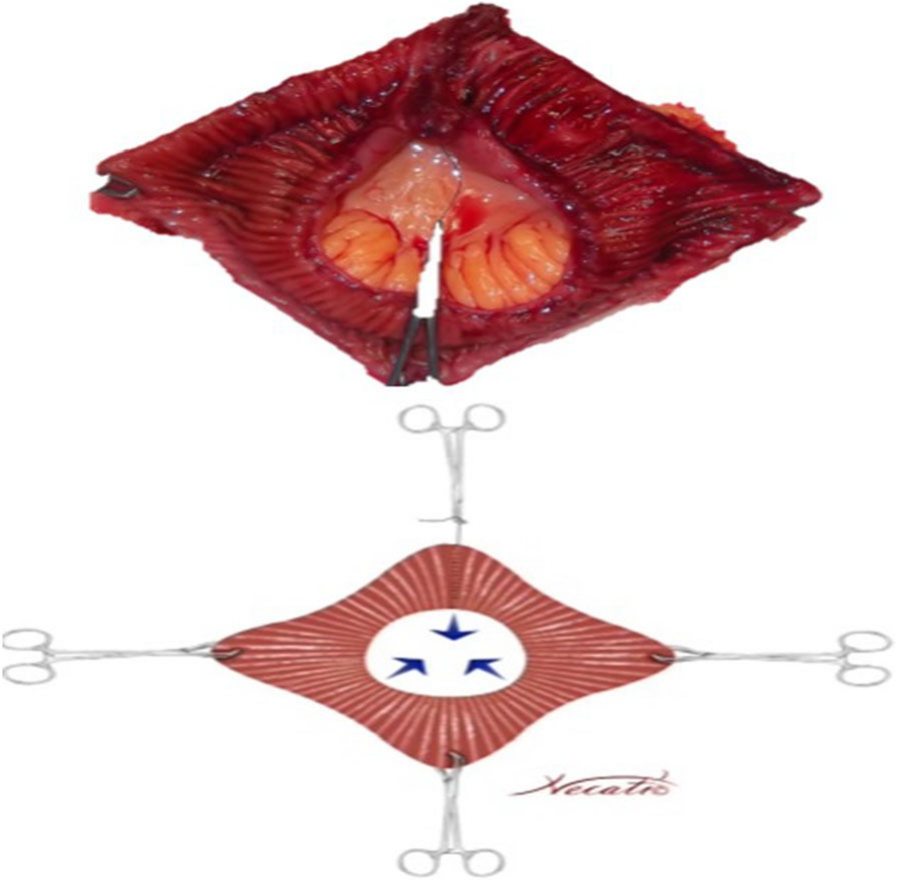
A bagel-shaped detubularized ileal segment is formed

**Fig. 5:**
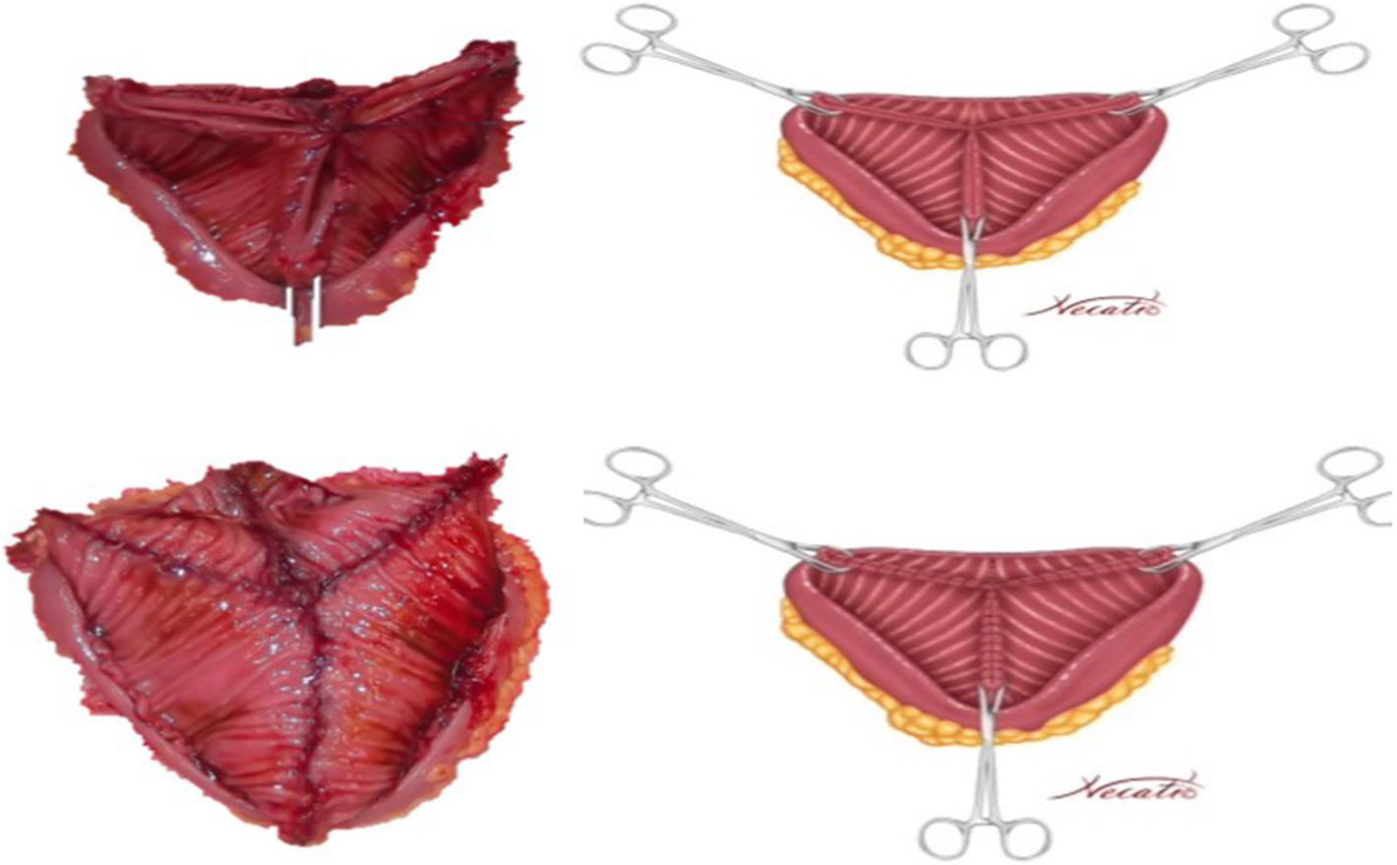
Three identical points, starting from the medial border of the anastomosis segment, are identified and united at the center, and the medial edges of the ileal loop are joined

**Fig. 6:**
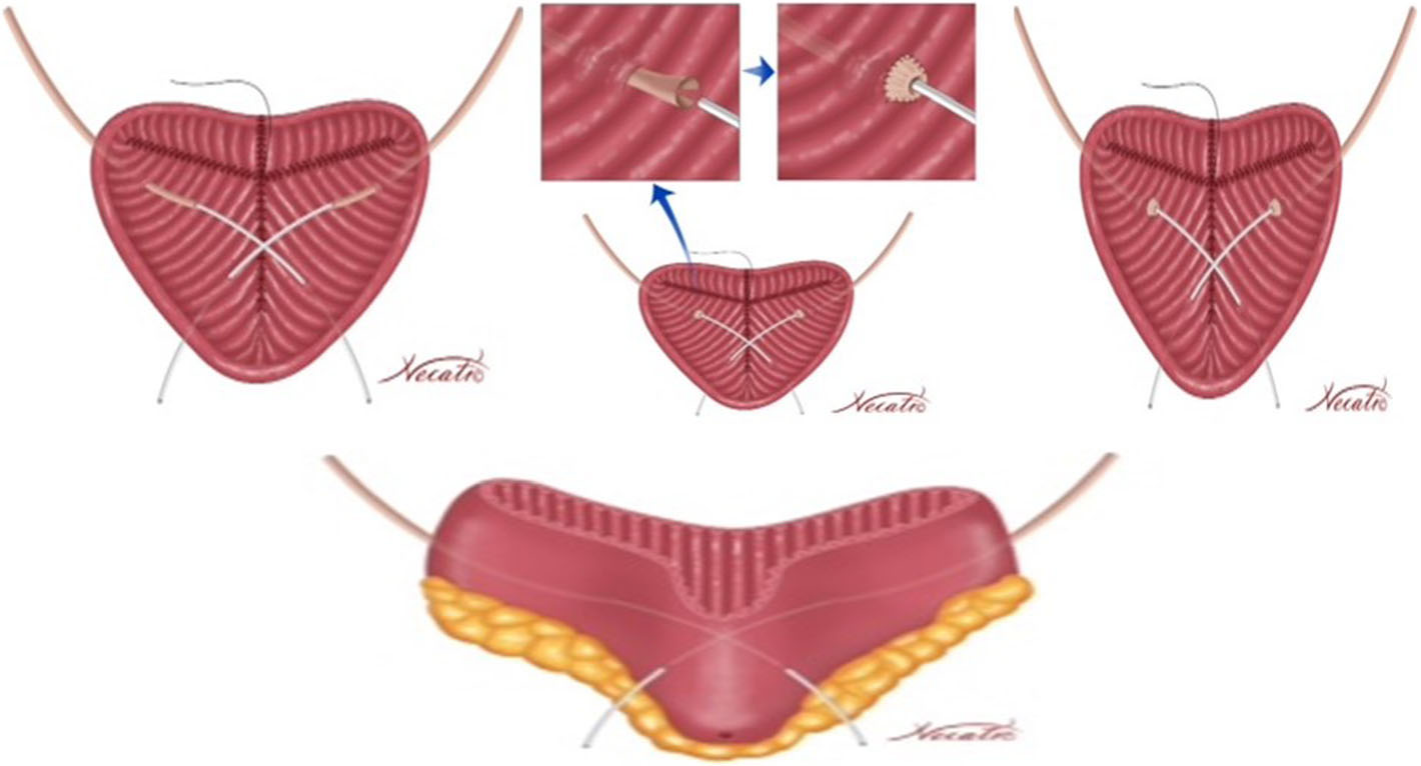
Both ureters are spatulated and anastomosed end-to-side to the neobladder over appropriate single J stents with an antireflux mechanism

**Fig. 7:**
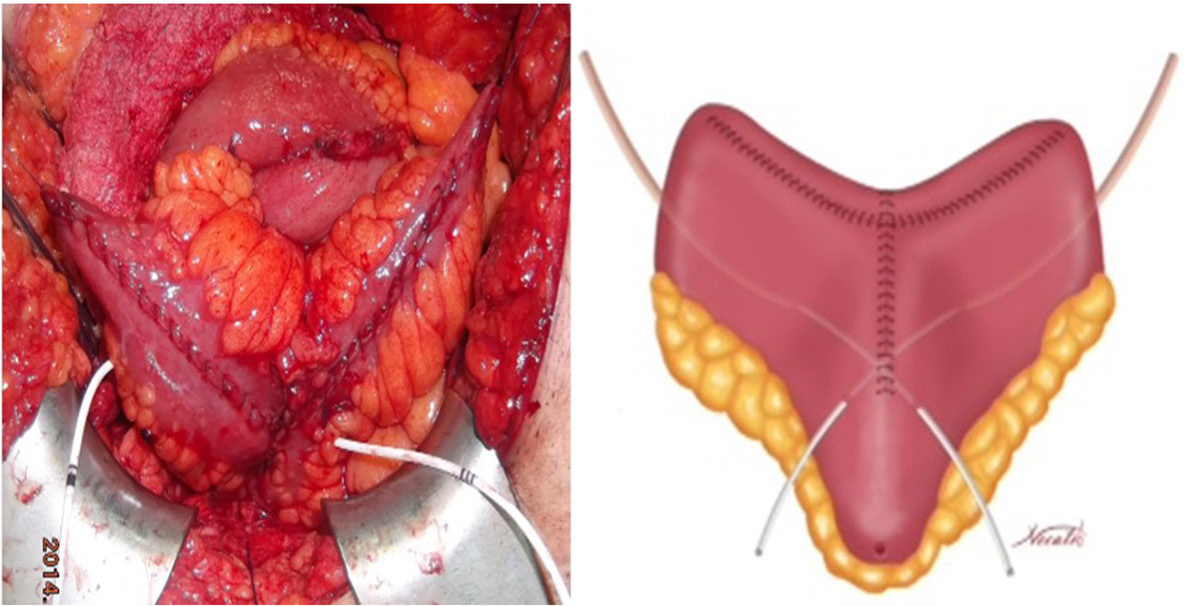
The triangular-shaped Anatolian pouch is formed. The ureteral stents are fixed to the ileal mucosa and taken out of the pouch by stabbing the anterior wall of the reservoir. The urethroileal anastomosis is performed over a transurethral 22-French catheter with six to eight 3–0 polyglactin sutures

A 45 cm ileal segment was separated, starting at an appropriate point 15 to 20 cm from the ileocecal junction to avoid gastrointestinal problems postoperatively (Fig. 1). The intestinal continuity was rebuilt by an end-to-end anastomosis with surgical staplers (Fig. 2). The whole separated ileal segment was cut at the antimesenteric border for detubularization. Proximal and distal sides of the ileal loop were anastomosed side-to-side and a bagel-shaped detubularized ileal segment was formed (Figs. 3 and 4). Three identical points, starting from the medial border of the anastomosis segment, were identified and united at the center. Then, the medial edges of the ileal loop were joined by a running through-and-through suture of 3–0 polyglactin continuously, resulting in a goosefoot image in the centrum (Fig. 5). After this stage, a triangular configuration was formed. Also, three points from the lateral side of the detubularized ileal loop were identified and united at the center, and the lateral edges of the ileal loop were sutured continuously with 3–0 polyglactin, leaving the lower part of the reservoir open for the urethroileal anastomosis. Both ureters were spatulated and anastomosed end-to-side to the neobladder over appropriate single J stents with an antireflux mechanism (Fig. 6). The ureteral stents were fixed to the ileal mucosa and taken out of the pouch by stabbing the anterior wall of the reservoir (Fig. 7). The urethroileal anastomosis was performed over a transurethral 22-French catheter with six to eight 3–0 polyglactin sutures. The pelvis was drained with a 28-French tube drain.

## Results

**Fig. 8:**
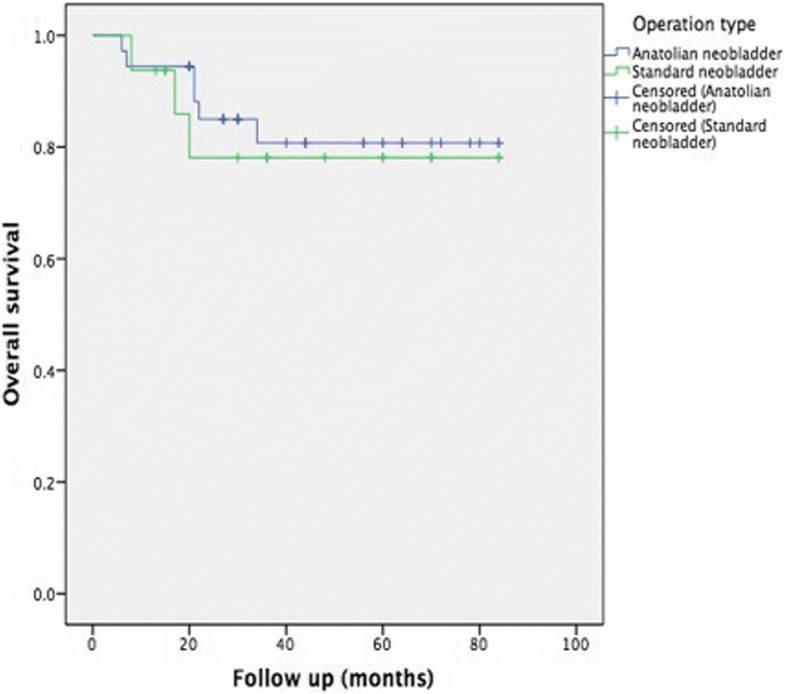
Kaplan Meier analysis of overall survival (OS)

Median follow-up was 44 months (range, 12–85 months). The operation was technically successful in all cases. There were no intraoperative or perioperative deaths. Mean operative time was 5.5 h (range, 4–6.5 h) and 5.6 h (4–7 h) for novel technique and standard technique, respectively (*p* < 0.001). There were no severe intraoperative complications. Mean amount of blood loss was 550 mL (350–1700 mL) and 580 mL (300–1800 mL), for novel technique and standard technique, respectively (*p* = 0.22). The perioperative and postoperative outcomes were given in Table 2. Kaplan Meier analysis of overall survival (OS) for two techniques was showed in Fig. 8.

**Table 2:** The perioperative and postoperative outcomes

	Novel technique (Anatolian Neobladder) (*n* = 36)	Standard technique (Studer neobladder) (*n* = 16)	*p*
Bleeding (ml)	550 (350–1700)	580 (300–1800)	0.22
Operation time (h)	5.5 (4–6.5)	5.6 (4–7)	< 0.001
Hospitalization time (day)	7.3 (6–20)	7.3 (5–14)	0.80
Early postoperative complications	12 (33.3%)	5 (31.2%)	0.55
-Urinary infection (Grade 2^a^)	5 (13.8%)	2 (12.5%)	
-Paralytic ileus (Grade 2^a^)	3 (8.3%)	1 (6.2%)	
-Skin infection (Grade 2^a^)	4 (11.1%)	2 (12.5%)	
Late postoperative complications	6 (16.6%)	3 (18.7%)	0.65
-Urinary infection	3 (8.3%)	1 (6.2%)	
-Incisional hernia	1 (2.7%)	–	
-Urethro-neobladder stenosis	1 (2.7%)	1 (6.2%)	
-Uretero-neobladder stenosis	1 (2.7%)	1 (6.2%)	
Survival time (months) (95%CI)	71.6 (62.6–80.6)	69.1 (53.9–84.1)	0.72
Perioperative admission rate	27.7%	25.0%	0.84
Postmictuonal residual urine (mL) 2. month	34.7	34.4	0.80
Postoperative ICIQ-SF score	2.7 (0–21)	2.6 (0–21)	0.72
Continence improved			
-Daytime continence	32/36 (88.8%)	14/16 (87.5%)	0.89
-Nighttime continence	20/36 (55.5%)	9/16 (56.2%)	0.96

^a^According to the Clavien classification of surgical complications

**Fig. 9:**
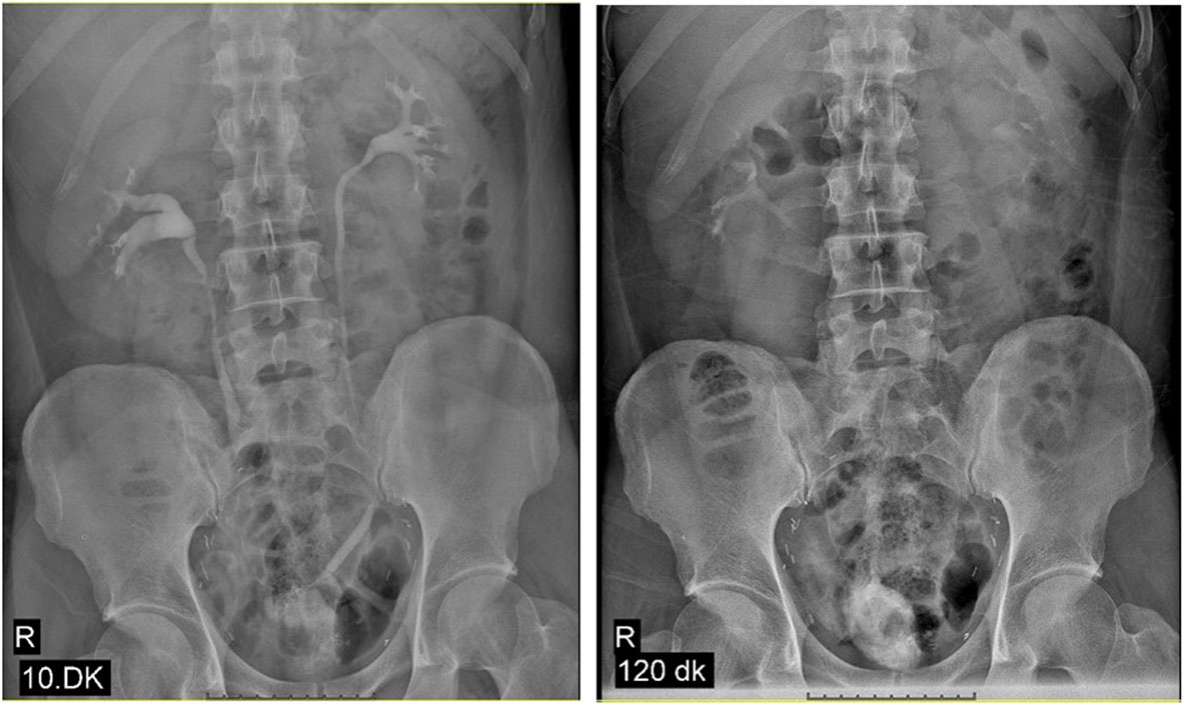
IVP shows no dilatation of the upper urinary tract system

Early postoperative complications were occurred in 33.3% and 31.2% of the patients, for novel technique and standard technique, respectively (*p* = 0.55). According to the Clavien classification of surgical complications, a Grade 2 urinary infection occurred in 5 patients (13.8%) and 2 patients (12.5%) for novel technique and standard technique, respectively, all of whom improved with antibiotic treatment. Grade 2 paralytic ileus occurred in 3 patients (8.3%) and 1 patient (6.2) for novel technique and standard technique, respectively; however, open surgery was not required in any patient. Late complications were noted in 6 patients (16.6%) and 3 patients for novel technique and standard technique, respectively; urinary infection in three (8.3%) for novel technique and 1 patient for standard technique, and incisional hernia in one (2.7%) for novel technique. The latter patient required additional surgical treatment. Stenosis of the ureterointestinal anastomosis in one patient (2.7%) for novel technique and 1 patient (6.2%) for standard technique were repaired with open surgery. There were no metabolic complications. An intravenous pyelogram (IVP) was performed when necessary (Fig. 9). Ureteral stenosis in one patient (2.7%) for novel technique and 1 (6.2%) for standard neobladder were treated endoscopically. The perioperative admission rate was found 27.7% and 25% for novel technique and standard technique, respectively. Six patients (16.6%) in novel technique and 3 patients (18.7%) in standard technique died during follow-up. Five of them in novel technique died because of recurrent bladder cancer and one of unrelated cancer. Also, 2 of them in novel technique died because of recurrent bladder cancer and one of unrelated cancer.

**Fig. 10:**
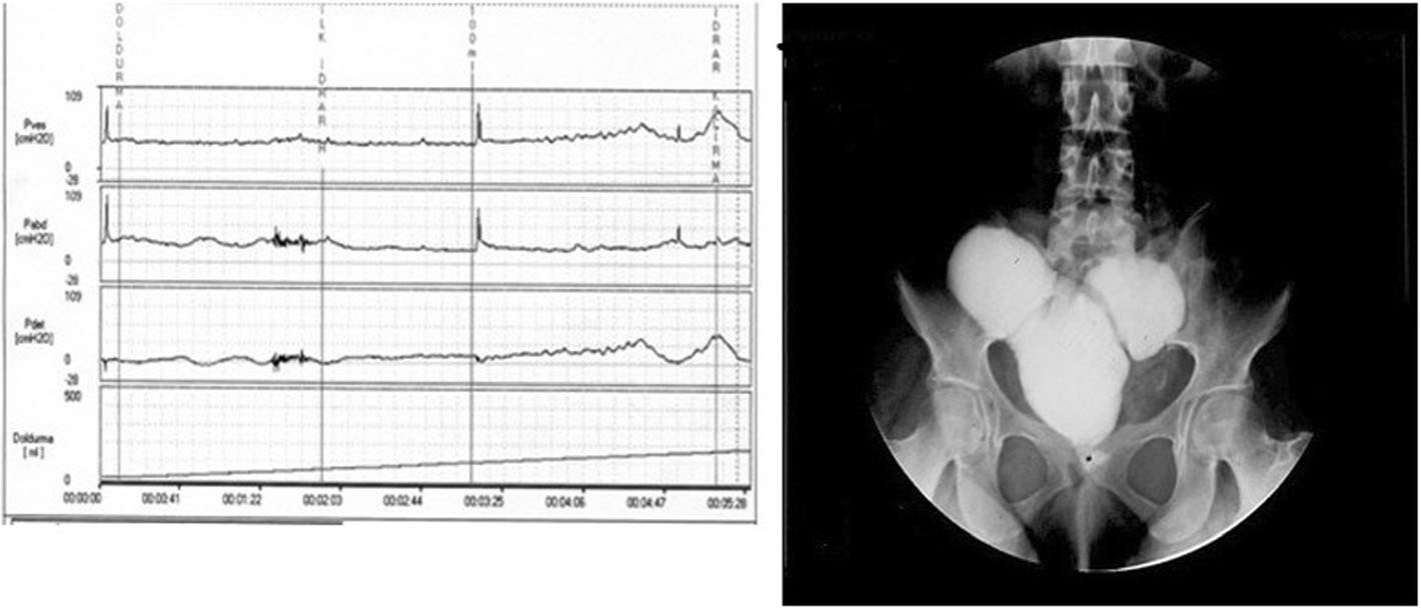
Urodynamic evaluation was performed in a patient with daytime incontinence during follow-up. The cystometric image also is seen in the Figure

Continence improved by stages with time and most patients became continent within a mean of 3.5 months (range, 1–12 months) postoperatively for both two techniques. Self-catheterization was performed before bedtime to improve nighttime continence until patients regained continence. Complete daytime continence was achieved in 32 of the 36 patients (88.8%) and 14 of the 16 patients (87.5%) for novel technique and standard technique, respectively (*p* = 0.89). Nighttime continence was achieved in 20 (55.5%) and 9 (56.2%) patients, for novel technique and standard technique, respectively (*p* = 0.96). Urodynamic evaluation was performed in four patients of novel technique (Fig. 10) and revealed urodynamic stress incontinence in two and low pressure low flow in two.

## Discussion

Various types of orthotopic neobladders are used as a method of urinary diversion after radical cystectomy. Although new neobladder techniques have been described, it is still controversial as to how best to shape it. A normal neobladder should be safe and easy to create. Ideally, it should have low pressure. It should also be of the appropriate capacity. There should be no reflux in the upper urinary tract and the development of stricture in the ureters should be prevented. In addition, day- and nighttime continence should be ensured [8]. The Studer neobladder and other ileo-colonic neobladder techniques which are known for long term outcomes are the most frequently performed methods [5, 6, 9–11]. The Studer technique has these characteristics and has significant advantages [6]. Generally, surgeons adopt one or two techniques that are suitable for them. As surgeons perform a significant number of these reconstructive techniques per year, it is reasonable for them to adopt a technique that is appropriate, easy to perform and long-term results are known. Our clinic is a high volume center for radical cystectomy (> 25) and a mean of 30 patients underwent this operation within a year. These reasons prompted us to design a new neobladder construction. In our novel technique, the operative time is acceptable. The main advantage of this technique is that a simple shape, like an original bladder configuration, is constructed. The ileal segment used for the ureteroileal anastomosis is formed spontaneously and the ureters are anastomosed to the bottom of the neobladder without creating a chimney (Fig. 7). Residual urine volume was not significant in patients due to the bladder configuration. In the Anatolian neobladder, we fixed both upper corners of the ileal pouch to the psoas muscles. In our opinion, this was another factor that led to good emptying of the neobladder.

The ureters were anastomosed to the neobladder according to the technique of Abol-Eneim and Ghoneim [12]. We anastomosed the spatulated end of the ureters to the intestinal mucosa using a direct mucosa-to-mucosa and inverted ureteral nipple technique. Use of an isoperistaltic limb of ileum as an antireflux mechanism offers the advantage of an easily constructed ureterointestinal anastomosis with a low incidence of reflux and confusion of the upper tracts. To date, of 72 renal units, there is no confusion of any renal unit. This rate of upper tract preservation is acceptable. Before removing the Foley catheters postoperatively, we routinely perform cystography to demonstrate no reflux in the renal units.

There were no severe intraoperative complications in our study. Mean blood loss was 550 mL (range, 350–1700) and 580 mL (300–1800 mL), for novel technique and standard technique, respectively and there were no statistically significant differences between two techniques (*P* = 0.22). Protection of the upper urinary tract is a critical point during orthotopic neobladder reconstructions. In our study, urinary infection occurred in eight patients (22.2%), all of whom improved with medical treatment. Rogers and Scardino used a modification of the Studer technique and reported acute pyelonephritis in two of 20 patients (10%) [13]. In another study, Yoneda et al. used a modified Studer technique and reported acute pyelonephritis in 27% of the patients [14]. Our series had a similar rate of pyelonephritis and there was no statistically difference for postoperative urinary infection rates between novel technique and standard neobladder in this study. Paralytic ileus occurred in three patients (8.3%) for novel technique and in 1 (6.2%) for standard neobladder, but open surgery was not required in any patient. Incisional hernia in one patient required additional surgery. No other late complications occurred. Stenosis of the uretero-neobladder anastomosis in one patient (2.7%) for novel technique and 1 patient for standard technique (6.2%), were treated by additional open surgery. Ureterointestinal anastomosis should prevent stenosis and obstruction [15]. Stenosis of the urethra in one patient (2.7%) for novel technique and 1 patient (6.2%) for standard technique, were treated endoscopically. Authors should remember that the rate of complications after radical cystectomy and orthotopic urinary diversion is not to be underestimated. In the review published by Faba et al., they stated that the complications were significant after cystectomy and orthotopic urinary diversion and they were not as low as in previous publications [16]. It was emphasized that complications were encountered in the 20th year after the operation and therefore follow-up should be done.

In our study, there were no metabolic complications. Use of the terminal ileum was not advocated because of the potential risk of vitamin B12 and bile acid malabsorption, and resultant diarrhea [17]. Exclusion of the ileocecal valve from the normal alimentary tract and interference with feces transit time also may account for diarrhea in these patients. There are several reports of metabolic acidosis occurring in patients during follow-up [5, 6]. Hautmann et al. reported that 48% of patients with an ileal neobladder required alkalizing treatment for acidotic imbalance [5]. Gakis et al. described the advantages of using a terminal ileal segment for orthotopic urinary diversion [17]. Metabolic consequences due to bowel wall secretion and urinary reabsorption from the intestinal reservoir can be compensated best in the terminal ileum or jejunum. As a result, the terminal ileal segment is the most ideally suited bowel segment for orthotopic urinary diversion. There were no metabolic complications in our patients during the early follow-up period. We routinely evaluated laboratory values and replaced vitamin B and sodium bicarbonate if necessary during long-term follow-up.

Complete daytime continence was achieved in 32 of the 36 patients (88.8%) and 14 of the 16 patients (87.5%) for novel technique and standard technique, respectively (*p* = 0.89). Also, nighttime continence was achieved in 20 (55.5%) and 9 (56.2%) patients, for novel technique and standard technique, respectively (*p* = 0.96) and there was no statistically significant difference between two techniques. Parekh et al. reported that patients with bladder substitution achieved daytime control more rapidly than those who underwent radical prostatectomy, and stress urinary incontinence was rare [18]. Also, patient adaptation and mental capacity to understand the new bladder are important factors for achieving continence. Our success rate for achieving continence was similar to that of other studies [5, 6, 13, 15]. In addition, there was no statistically difference between our novel technique and standard technique in this study.

Finally, the complication rate was acceptable, and there was no perioperative mortality for novel technique and standard technique, in this study. In addition, this bladder substitute of novel technique appears to be technically easier and safe. However, there are several limitations to our study. One limitation was that our data were collected retrospectively. Consecutively, 36 patients were included in this study and compared with 16 patients who underwent standard neobladder. However, the number of patients was limited and long-term results were not known. This might have decreased the power of the study. However, the functional results and postoperative morbidity rates in our series were acceptable. Future studies including larger series of patients should be designed prospectively to overcome existing limitations.

## Conclusions

Our study demonstrated the feasibility of novel technique (Anatolian neobladder) in the treatment of bladder cancer after radical cystectomy. It can be an alternative to other ileal neobladder techniques. Further prospective and randomized controlled comparative studies including large series of patients are needed.

## Abbreviations

CT: Computerized tomography
ICIQ-SF: International Consultation on Incontinence Questionnaire Short Form
OS: Overall survival
